# Positive end-expiratory pressure increases intracranial pressure but does not affect PRx, regardless of body position, in a porcine ARDS model

**DOI:** 10.3389/fphys.2026.1792273

**Published:** 2026-03-25

**Authors:** Rønnaug Hammervold, Erta Beqiri, Peter Smielewski, Benjamin S. Storm, Erik W. Nielsen, Claude Guérin, Shirin K. Frisvold

**Affiliations:** 1 Department of Anaesthesia and Intensive Care, Nordland Hospital Trust, Bodø, Norway; 2 Department of Clinical Medicine, UiT The Arctic University of Norway, Faculty of Health Sciences, Tromsø, Norway; 3 Research Laboratory, Nordland Hospital Trust, Bodø, Norway; 4 Brain Physics Laboratory, Department of Clinical Neurosciences, University of Cambridge, Cambridge, United Kingdom; 5 Nord University, Faculty of Nursing and Health Sciences, Bodø, Norway; 6 University of Oslo, Faculty of Medicine, Institute of Clinical Medicine, Oslo, Norway; 7 Université de Lyon, Faculté de médecine Lyon-Est, Lyon, France; 8 Department of Anaesthesia and Intensive Care, University Hospital of North Norway, Tromsø, Norway

**Keywords:** ARDS, cerebral autoregulation, cerebral monitoring, intracranial pressure, mechanical ventilation, prone position, respiratory mechanics

## Abstract

**Background:**

Positive end-expiratory pressure (PEEP) and prone positioning are key components in the management of acute respiratory distress syndrome (ARDS), improving gas exchange and protecting the lung via enhanced lung recruitment and homogenization of lung aeration. However, higher intrathoracic pressures may increase intracranial pressure (ICP) or impair cerebral autoregulation, as reflected by vascular reactivity. This study investigated whether stepwise PEEP elevations affect ICP, the pressure reactivity index (PRx), and brain tissue oxygenation (PbO2) in a porcine ARDS model, comparing prone and supine positions, and whether baseline physiological variables modify the ICP response.

**Methods:**

Twelve anesthetized pigs with bronchial lavage–induced ARDS were studied in a randomized crossover design with stepwise PEEP increases (5, 10, 15, and 20 cmH2O) in prone and supine positions while maintaining stable arterial carbon dioxide tension and cerebral perfusion pressure. Intracranial pressure, PRx, and PbO2 were continuously monitored, and effects of PEEP and position were analyzed using linear mixed-effects models and analysis of variance.

**Results:**

Increasing PEEP was associated with a progressive rise in ICP, whereas PRx remained unchanged across PEEP levels and body positions. PbO2 showed a non-significant upward trend with increasing PEEP, while the PbO2/PaO2 ratio remained stable. Higher baseline pulmonary artery pressure was associated with larger ICP increases, whereas higher baseline respiratory rate was associated with attenuated responses.

**Conclusion:**

In this porcine ARDS model, moderate PEEP escalation resulted in a modest increase in ICP without impairment of cerebrovascular reactivity, with similar effects in prone and supine positions, suggesting that lung protective ventilation strategies may be compatible with stable intracranial physiology when arterial carbon dioxide tension and cerebral perfusion pressure are controlled.

## 1 Introduction

Sufficient positive end-expiratory pressure (PEEP) is a cornerstone in the management of acute respiratory distress syndrome (ARDS) (Fan et al., 2018; Sweet et al., 2023; Qadir et al., 2024). Prone positioning and individualized PEEP titration, potentially guided by transpulmonary pressure (TPP), are important strategies within this approach (Gattinoni et al., 2013; Akoumianaki et al., 2014; Mezidi et al., 2018; Guérin et al., 2020).

However, increasing PEEP raises intrathoracic pressure, which may reduce venous return and cardiac output, potentially elevating intracranial pressure (ICP) or compromising cerebral perfusion (Mrozek et al., 2015; Matin et al., 2022; Ziaka and Exadaktylos, 2022; Asehnoune et al., 2023; Taran et al., 2023). This interaction is clinically relevant as ARDS and acute brain injury (ABI) frequently coexist. Evidence on the effects of PEEP on ICP is conflicting, with responses varying according to lung compliance, baseline ICP, and arterial carbon dioxide (PaCO_2_) levels (Beqiri et al., 2023; Bencze et al., 2025; Mascia et al., 2025). Prone positioning not only improves oxygenation and reduces mortality in moderate-to-severe ARDS but may also influence intrathoracic pressure transmission and thereby affect intracranial physiology (Elmaleh et al., 2024).

While increasing PEEP and using prone position are aimed at improving systemic oxygenation, little is known about their effects at the brain oxygenation level. Monitoring of brain tissue oxygenation (PbO_2_) provides additional information on cerebral oxygen delivery and may help detect cerebral hypoxia that is not apparent when relying solely on ICP and cerebral perfusion pressure (CPP) monitoring (Chesnut et al., 2020; Robba et al., 2020b), although evidence of its clinical utility remains mixed (Bernard et al., 2022; Jeffcote et al., 2024).

Cerebral autoregulation (CA) reflects the brain’s ability to maintain stable perfusion despite fluctuations in CPP. Continuous monitoring of CA may offer complementary information to ICP monitoring alone (Casault et al., 2022) in the management of brain injury patients. The pressure reactivity index (PRx) is a validated metric of CA, where positive values indicate impaired autoregulation (Howells et al., 2005; Zweifel et al., 2008) and are associated with worse outcomes in traumatic brain injury. Few studies have examined the relationship between ventilator pressures and PRx. One clinical study (Beqiri et al., 2023) of patients with healthy lungs indicates that PRx remains relatively stable during moderate elevations in PEEP if cerebral compliance is preserved; however, evidence from ARDS populations remains scarce.

Similarly, in our previous study in healthy pigs, increasing PEEP elevated ICP but did not impair PRx in either prone or supine position (Hammervold et al., 2024). Whether these findings remain valid in ARDS remains unclear.

The aim of this study was to investigate how changes in PEEP in the prone and supine positions affect ICP and PRx in a porcine model of ARDS with normal baseline ICP. By integrating measurements of TPP, PbO_2_, and advanced ICP waveform features, we aim to elucidate the cerebral, respiratory, and hemodynamic effects of PEEP under ARDS conditions.

## 2 Materials and methods

This prospective, randomized, controlled animal study was approved by The Norwegian Animal Research Authority (FOTS ID 27107). The study complied with the Norwegian Laboratory Animal Regulations and the EU Directive 2010/63/EU. Reporting followed the ARRIVE guidelines. The methodological setup was largely based on our previously published protocol in healthy pigs (Hammervold et al., 2024), with the addition of an ARDS model described below.

### 2.1 Animal preparation

Fifteen Norwegian Landrace pigs (Sus scrofa domesticus) were included. Three animals were excluded due to poor ICP signal quality (n = 1), refractory hypotension (n = 1), and pneumothorax (n = 1), resulting in a final sample of 12 pigs (11 males, 1 female) with a mean weight of 25 kg (range 23–28 kg). All animals underwent ICP monitoring, and a subset of eight animals also received PbO_2_ monitoring, as probes were unavailable in two animals and technical failure occurred in two despite correct placement.

Animals were sedated with intramuscular ketamine (20 mg/kg), midazolam (0.5 mg/kg), and atropine (1 mg) before transport to the North University of Bodø animal research facility. Anesthesia was maintained via a peripheral venous access using continuous intravenous morphine (2 mg/kg/h), midazolam (0.15 mg/kg/h), and pentobarbital (4 mg/kg/h). Animals were intubated in the prone position using a 6.5 mm endotracheal tube (Portex^®^, Smiths Medical, Minneapolis, MN, USA), and anesthesia depth was verified via the pedal withdrawal reflex to ensure adequate anesthesia.

Mechanical ventilation was initiated in volume control mode using an Engström Carestation ventilator (GE Healthcare, Chicago, IL, USA) with a tidal volume (VT) of 9 mL/kg, fraction of inspired oxygen (FiO_2_) 0.40, PEEP 5 cmH_2_O, inspiratory:expiratory (I:E) ratio of 1:2, and inspiratory pause of 20%. Respiratory rate was adjusted to maintain PaCO_2_ between 35–45 mmHg. Esophageal pressure (Pes) was measured with a balloon catheter (FluxMed, MBMED, Buenos Aires, Argentina). Correct position of the balloon was verified with the Baydur occlusion maneuver (end-expiratory airway occlusion), confirming closely matched changes in esophageal and airway pressures (ΔPes/ΔPaw = 0.8–1.2) (Baydur et al., 1982).

A 4 Fr pulse contour cardiac output (PiCCO) catheter (Pulsion Medical Systems, Getinge, Feldkirchen, Germany) was inserted into the left femoral artery using a percutaneous ultrasound-guided technique, and a Swan-Ganz catheter (Edwards Lifesciences, Irvine, CA, USA) via the right external jugular vein for pulmonary artery pressure (PAP) and cardiac output (CO) measurements. All catheters were connected to pressure transducers (TruWave, Edwards Lifesciences, Irvine, CA, USA) and the monitoring system (IntelliVue MP70, Philips Healthcare, Eindhoven, The Netherlands). Fluid management included an initial 500 mL bolus of Ringer acetate (B. Braun, Melsungen, Germany) followed by continuous infusion at 5 mL/kg/h. A suprapubic catheter was inserted for urinary drainage, using open surgical technique through a minilaparotomy. Blood temperature was continuously measured via the PiCCO catheter, and normothermia (38–39 °C) was maintained with active thermal management.

### 2.2 Induction of ARDS

ARDS was induced using a two-step model: surfactant depletion (Russ et al., 2016; Terzi et al., 2020) by saline lavage followed by injurious mechanical ventilation (Russ et al., 2021). Animals were ventilated in the supine position with FiO_2_ 1.0, PEEP 5 cmH_2_O, and VT 9 mL/kg. Warmed saline (30 mL/kg) was instilled via the endotracheal tube into the lungs by gravity and drained passively by positioning the pig in steep Trendelenburg. Lavage was repeated until the arterial oxygen pressure/fraction of inspired oxygen ratio (PaO_2_/FiO_2_) fell below 150 mmHg or until nine cycles had been completed, after which the animal was turned prone for injurious ventilation.

The injurious ventilation protocol involved FiO_2_ 1.0, PEEP 0 cmH_2_O, peak inspiratory pressure 40 cmH_2_O, respiratory rate 12/min, and I:E ratio 1:1.5, with VT limited to 30 mL/kg. ARDS was confirmed by PaO_2_/FiO_2_ <150 mmHg and a ≥30% reduction in respiratory system compliance.

This model reproduces key physiological features of ARDS but does not represent the full clinical syndrome (Zhao et al., 2025).

### 2.3 Cerebral monitoring

During the injurious ventilation phase, a burr hole was made in the skull, and a Licox^®^ Dual lumen bolt (Integra LifeSciences, Princeton, NJ, USA) was placed in the left frontal hemisphere. Through the bolt, an intraparenchymal Codman Microsensor^®^ ICP Transducer (Medos International SÀRL, Le Locle, Switzerland) and a Licox^®^ brain tissue oxygen probe (Integra LifeSciences) were inserted into the left frontal white matter. Signals were recorded via the Licox^®^ Monitor (Integra Life Sciences) and Codman ICP Express (Codman & Shurtleff Inc., Raynham, MA, USA) and integrated with the IntelliVue MP70 monitor.

### 2.4 Physiological monitoring and data acquisition

Continuous monitoring included ECG, arterial oxygen saturation (SpO_2_), end-tidal carbon dioxide (EtCO_2_), body temperature, arterial blood pressure (ABP), central venous pressure (CVP), and PAP. CO, extravascular lung water index (EVLW), and stroke volume variation (SVV) were calculated by the PiCCO/Swan–Ganz systems. ICP, PbO_2_, and all ventilator-derived parameters were collected continuously. All signals were streamed to a laptop with ICM+^®^ software (Cambridge Enterprise Ltd, Cambridge, UK) for synchronized, high-resolution data integration, recording, and storage. At each step of PEEP titration, arterial blood gases were sampled to assess pH, PaO_2_, and PaCO_2_.

### 2.5 Study procedure

**Figure 1:**
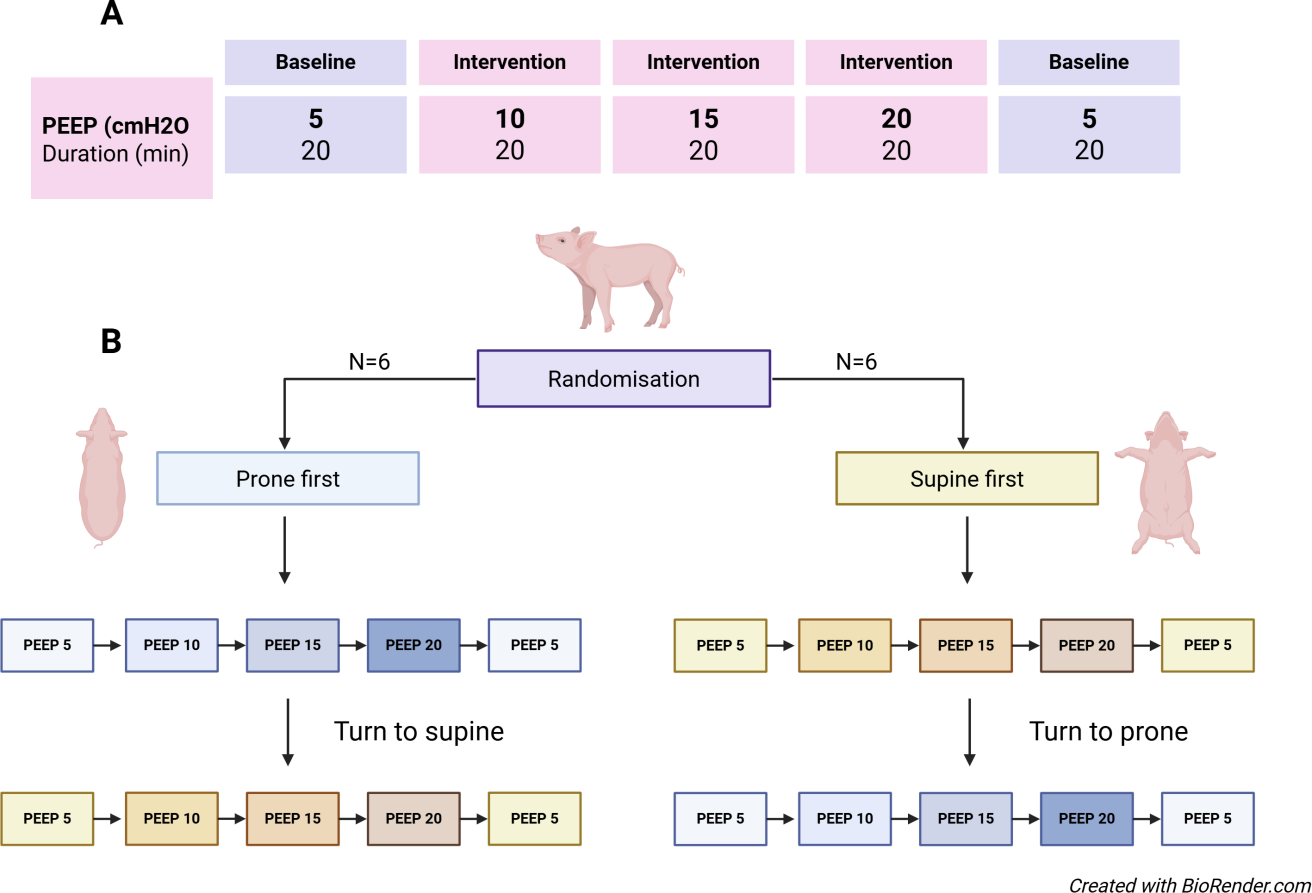
Study design: crossover design and PEEP protocol. **(A)** Illustrates the stepwise PEEP sequence and duration for each study period. During baseline, animals were ventilated with a tidal volume of 9 mL/kg body weight and a positive end-expiratory pressure of 5 cmH_2_O. Subsequent measurements were performed at PEEP levels of 5, 10, 15, and 20 cmH_2_O, each maintained for 20 minutes, with ventilation adjusted to keep arterial PaCO_2_ stable. **(B)** Depicts the randomization and crossover sequence. In the prone–supine group (n = 6), the PEEP trial was performed first in the prone position and repeated in the supine position. In the supine–prone group (n = 6), the sequence was reversed. PEEP, positive end-expiratory pressure; PaCO_2_, arterial carbon dioxide tension.

The crossover design, randomization scheme, and PEEP protocol are illustrated in **Figure 1**.

Randomization by drawing lots was performed prior to the experiment by personnel not involved in data collection. Each animal started in either the prone or supine position, followed by crossover to the opposite position. The PEEP protocol was identical in both positions, increasing stepwise from 5 to 10, 15, and 20 cmH_2_O, with each level maintained for 20 minutes before returning to baseline (PEEP 5 cmH_2_O).

Ventilator settings during the PEEP trial were FiO_2_ 0.40, I:E ratio 1:2, and a 20% inspiratory pause. Respiratory mechanics were assessed by rapid airway occlusion technique, and this was followed by thermodilution measurements at each PEEP level. To maintain PaCO_2_ as stable as possible, VT and RR were adjusted as needed and verified by arterial blood gases at each PEEP step. Euvolemia and a cerebral perfusion pressure (CPP) above 60 mmHg were maintained with intravenous fluids and norepinephrine as required.

### 2.6 Data processing

**Figure 2:**
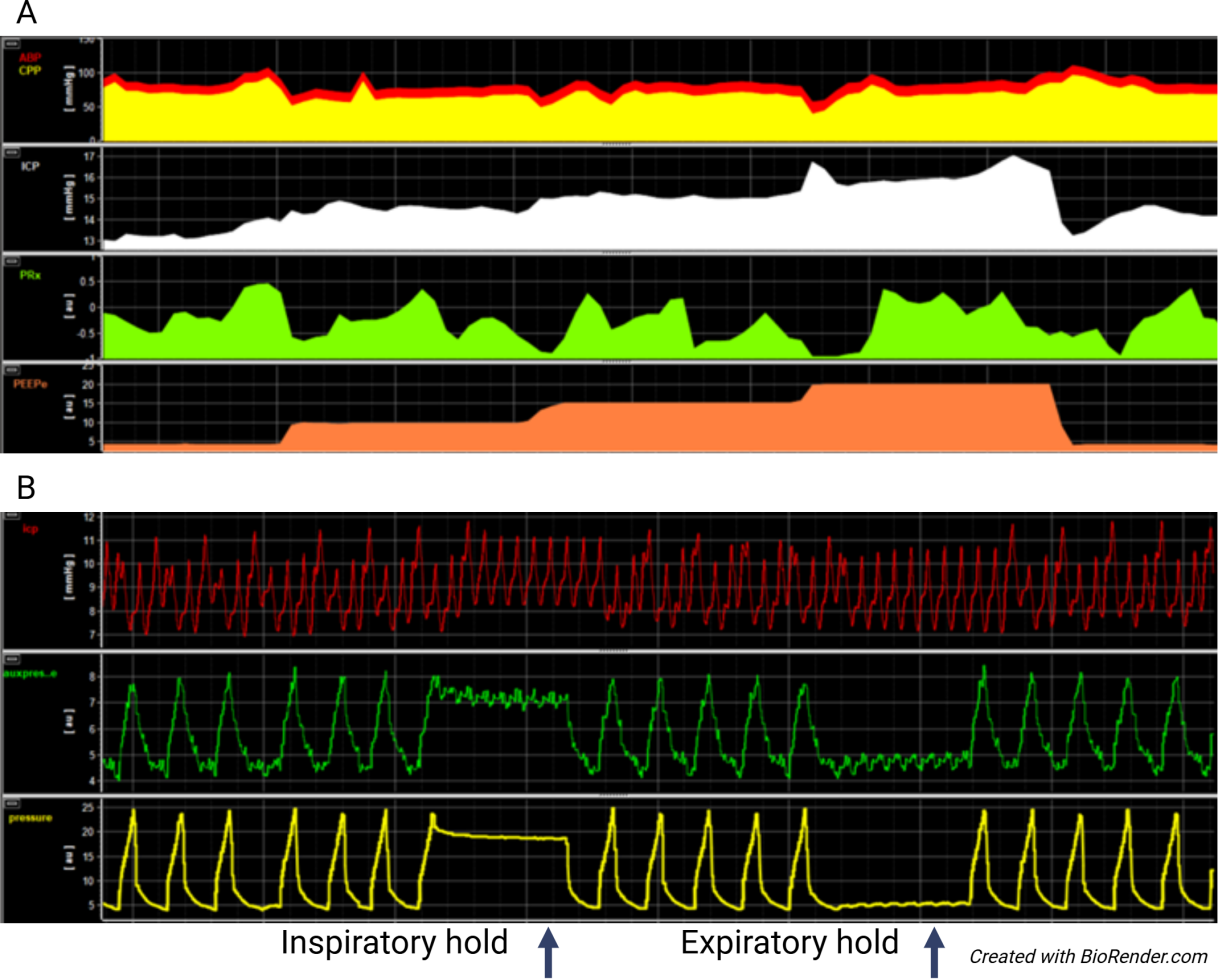
Example of multimodal physiological data acquisition and analysis using ICM+ software. **(A)** Displays averaged (mean) time-trend data illustrating cerebral and respiratory monitoring, including arterial blood pressure (ABP), cerebral perfusion pressure (CPP), intracranial pressure (ICP), pressure reactivity index (PRx), and positive end-expiratory pressure (PEEP). **(B)** Displays high-resolution raw data traces showing the original waveform signals used for subsequent averaging and analysis. Shown variables include intracranial pressure (ICP), esophageal pressure (auxpressure), and airway pressure (pressure). The panel also illustrates inspiratory and expiratory airway and esophageal occlusion maneuvers, with duration of 5 seconds.

ICM+^®^ software was used to preprocess high-resolution recordings prior to statistical analysis (Smielewski et al., 2024) (**Figure 2**). Two researchers (RH and SF) independently reviewed each recording to assess signal quality and visually inspect data for artifacts. Major artifacts were first removed manually, after which automated artifact detection was applied in ICM +. ICP values below –10 mmHg or above 60 mmHg were rejected, as were CPP values below 0 mmHg or above 150 mmHg.

All signals were then downsampled to 0.1 Hz by averaging non-overlapping 10-second segments (coarse graining). The pressure reactivity index (PRx) was calculated as a moving Pearson correlation between 30 consecutive 10-second averages of ABP and ICP (corresponding to 5 minutes), updated every minute. The compensatory reserve index (RAP) was computed in the same manner using intracranial pulse amplitude (AMP) and ICP (Klein et al., 2024; Smielewski et al., 2024).

Before statistical analysis, PRx and RAP values were Fisher-transformed. For each study period, descriptive statistics were exported for all variables using the event tool in ICM+, and mean values were used for subsequent analysis.

### 2.7 Respiratory mechanics variables

Respiratory mechanics data were exported using the ICM+^®^ software as one value per period.

End-inspiratory (Pawei) and end-expiratory airway (Pawee) and esophageal (Pesei and Pesee, respectively) pressures were recorded at zero flow after 5-second end-inspiratory and 5-second end-expiratory holds. Peak pressures of the respiratory system (Ppeakrs) and the chest wall (Ppeakcw) were defined as the maximal Paw and Pes values, respectively. RR and VT were also collected.

Peak transpulmonary pressure (Ppeakl), respiratory system elastance (Ers), chest wall elastance (Ecw), lung elastance (El), end-expiratory transpulmonary pressure (TPPee), end-inspiratory transpulmonary pressure (TPPei), elastance-derived transpulmonary pressure (TPPelast), driving pressure (DP), and mechanical power variables (MPrs, MPlDep, MPlnonDep) were computed according to standard formula (**Supplementary Table 1**).

### 2.8 Statistical analysis

The following variables were included in the statistical analysis based on their physiological relevance and in line with our previous study on healthy lungs (Hammervold et al., 2024), allowing direct comparison:

- Cerebral and hemodynamic variables: ICP, PRx, RAP, ABP, CPP, CVP, PAP, CO, PbO_2_, and temperature.
- Gas exchange and respiratory variables: SpO_2_, PaCO_2_, PaO_2_, pH, PaO_2_/FiO_2_ ratio, RR, Pawei, PEEPtot, Pesei, Pesee, TPPei, TPPee, TPPelast, Ers, Ecw, El, MPrs, MPlDep, MPlnonDep, EVLW, SVV, PEEP, VT, Ppeakrs, Ppeakcw, and Ppeakl.

Normality of continuous variables was assessed using the Shapiro–Wilk test. Homogeneity of variance was evaluated using Levene’s and Bartlett’s tests. Depending on normality, data were reported as mean ± standard deviation (SD) or median with interquartile range (IQR).

### 2.9 Statistical analysis strategy

Assumptions of the crossover design (absence of carry-over, sequence, and period effects) were tested for ICP and PRx using linear mixed-effects models. The sequence variable represented the order of positioning (prone–supine vs. supine–prone), and the period variable corresponded to the applied PEEP level (5, 10, 15, or 20 cmH_2_O). PEEP level, sequence, and period were the variables with fixed effects, with animal and position as those with a random effect.

#### 2.9.1 Primary endpoints

Baseline differences between prone and supine positions were tested using a two-sample t-tests for normally distributed variables and Mann–Whitney U tests for non-normal variables.

A two-way ANOVA was used to test the effects of PEEP level, body position, and their interaction on ICP and PRx. When no interaction was found, data from prone and supine positions were pooled and analyzed using repeated-measures ANOVA. Normality and homogeneity assumptions were checked; Welch’s ANOVA or Kruskal–Wallis tests were applied when assumptions were violated.

#### 2.9.2 Secondary endpoints

Secondary analyses examined the effect of PEEP and position on PbO_2_ and PbO_2_/PaO_2_ ratios. Finally, exploratory associations between changes in ICP and predefined baseline variables (e.g., ICP, CVP, Ers, TPPei, TPPee, RAP, PaCO_2_) were conducted using linear mixed-effects models. In line with our previous study (Hammervold et al., 2024), no multiple comparisons correction was applied because variables were selected *a priori* based on clinical relevance: PaO_2_ and PaCO_2_ were included for their influence on cerebral oxygenation and vasoreactivity; notably, PaCO_2_ is a potent regulator of cerebral blood flow and thereby of ICP. Pawei, TPPei, TPPee, Ers, and El were considered because they may modulate transmission of thoracic pressures to the cranial compartment. Likewise, CVP and PAP were assessed given their roles in venous return and pulmonary hemodynamics, which can affect cerebral venous drainage and ICP. MPrs, VTinsp, and RR were included to capture the mechanical energy delivered to the respiratory system, potentially shaping PEEP-related effects on the brain. Finally, CO was examined for its impact on cerebral blood flow, recognizing that PEEP can reduce venous return and cardiac output.

As a secondary descriptive analysis, ICP and respiratory responses to increasing PEEP were compared with our previous study in healthy lungs [24], which applied an identical crossover protocol in prone and supine positions. No formal between-study statistical testing was performed.

All statistical analyses were performed in R version 4.3 (R Core Team, 2021).

## 3 Results

### 3.1 Baseline characteristics

**Table 1** summarizes the baseline measurements recorded at PEEP 5 cmH_2_O in both prone and supine positions, prior to the stepwise PEEP increase.

**Table 1:** Baseline physiological values for study animals.

Variable	All animals (n=12)		
	Prone (n=6)	Supine (n=6)	p
Cerebral variables			
ICP (mmHg)	9 ± 7	12 ± 6	0.54
PRx	-0.084 ± 0.176	-0.049 ± 0.188	0.75
RAP	-0.043 ± 0.198	0.130 ± 0.145	0.11
CPP (mmHg)	83 (73-91)	75 (71-82)	0.33
PbO_2_ (mmHg)	22 (22-25)	28 (22-73)	0.79
Respiratory variables			
PaCO_2_ (mmHg)	50 ± 6	46 ± 7	0.23
PaO_2_ (mmHg)	140 (106-190)	71 (68-79)	0.06
PaO_2_/FiO_2_ (mmHg)	186 (159-205)	103 (76-134)	0.09
VTinsp (mL)	188 (183-199)	206 (185-220)	0.48
RR (breaths/min)	35 (26-36)	36 (28-40)	0.63
Ppeakrs (cmH_2_O)	31 (28-33)	33 (29-38)	0.33
Ppeakcw (cmH_2_O)	11 (10-13)	13 (11-16)	0.11
Ppeakl (cmH_2_O)	21 (18-22)	19 (16-26)	0.81
Pawei (cmH_2_O)	21 (19-24)	24 (22-27)	0.14
Pawee (cmH_2_O)	5 (5-6)	6 (5-6)	0.83
TPPei (cmH_2_O)	13 (10-15)	14 (11-17)	0.95
TPPee (cmH_2_O)	0.3 (-0.2-0.6)	-1.3 (-1.7- -1)	**0.04**
TPPelast (cmH_2_O)	17 (15-19)	20 (18-23)	0.07
Pesei (cmH_2_O)	8 ± 3	11 ± 2	0.11
Pesee (cmH_2_O)	5 ± 3	8 ± 2	0.07
Ers (cmH_2_O/L)	82 ± 14	92 ± 11	0.24
Ecw (cmH_2_O/L)	17 ± 4	17 ± 7	0.88
El (cmH_2_O/L)	65 ± 13	75 ± 7	0.13
DP (cmH_2_O)	18 ± 3	22 ± 5	**0.05**
MPrs (J/min)	14 ± 8	18 ± 9	0.48
MplDep (J/min)	9 ± 4	10 ± 7	0.73
MplnonDep (J/min)	8 ± 4	9 ± 6	0.71
Hemodynamic variables			
ABP (mmHg)	91 ± 12	87 ± 7	0.47
CVP (mmHg)	12 ± 7	15 ± 6	0.39
PAP (mmHg)	29 ± 6	32 ± 9	0.53
CO (L/min)	2.7 (2.6-2.9)	3.2 (3.1-3.4)	0.24
EVLW (mL)	485 (427-520)	529 (336-644)	0.87
SVV (%)	9 ± 2	10 ± 4	0.77
Systemic variables			
Body temperature (°C)	38.8 ± 0.5	38.6 ± 0.2	0.26

**Table 1** Values are presented as mean ± SD or median (IQR). p-values compare the prone and supine groups (two-sample t-test for normally distributed variables and Mann–Whitney U test for non-normally distributed variables). P5: Animals starting interventions in prone, positive end-expiratory pressure 5 cmH_2_O. S5: Animals starting interventions in supine, positive end-expiratory pressure 5 cmH_2_O. ICP, intracranial pressure; PRx, pressure reactivity index; RAP, compensatory reserve index; CPP, cerebral perfusion pressure; PbO_2_, brain tissue oxygenation; PaCO_2_, arterial partial pressure of carbon dioxide; PaO_2_, arterial partial pressure of oxygen; PaO_2_/FiO_2_, oxygenation index; VTinsp, inspiratory tidal volume; RR, respiratory rate; Ppeakrs, peak inspiratory pressure respiratory system; Ppeakcw, chest wall; Ppeakl, lung; Pawei/Pawee, end-inspiratory/-expiratory airway pressure; TPPei/TPPee, end-inspiratory/-expiratory transpulmonary pressure; TPPelast, elastance-derived transpulmonary pressure; Pesei/Pesee, esophageal pressure; Ers, elastance of the respiratory system; Ecw, chest wall; El, lung; DP, driving pressure; MPrs, mechanical power respiratory system; MPlDep, dependent lung; MPlnonDep, non-dependent lung; ABP, arterial blood pressure; CVP, central venous pressure; PAP, pulmonary artery pressure; CO, cardiac output; EVLW, extravascular lung water; SVV, stroke volume variation. Bold values indicate statistically significant differences.

Mean ICP at baseline was slightly higher in supine compared to prone (12 ± 6 mmHg vs. 9 ± 7 mmHg), though this difference was not statistically significant (p = 0.54). No significant differences were observed in PRx, RAP, ABP, CPP and PaCO_2_.

Among baseline respiratory variables, driving pressure was slightly higher in the supine group (22 ± 5 cmH_2_O) compared to the prone group (18 ± 3 cmH_2_O), showing a borderline significant difference (p = 0.05). Only TPPee differed slightly between positions (median -1.3 cmH_2_O in supine vs. 0.3 cmH_2_O in prone; p = 0.04). Oxygenation tended to be reduced in the supine group, with lower PaO_2_/FiO_2_ ratios (103 (76–134) vs. 186 (159–205) mmHg, p = 0.09). Notably, this was not accompanied by a lower PbO_2_ in supine, as PbO_2_ values remained stable between groups (28 (22–73) vs. 22 (22–25) mmHg, p = 0.79).

### 3.2 Effects of PEEP on ICP

Assumptions of the crossover design were met for the primary outcomes, with no significant carryover, sequence, or period effects for changes in ICP. There was no significant interaction between PEEP and body position (p = 0.75), indicating that the effect of PEEP on ICP was independent of prone or supine position. Similarly, position alone did not significantly affect ICP (p = 0.64).

**Figure 3:**
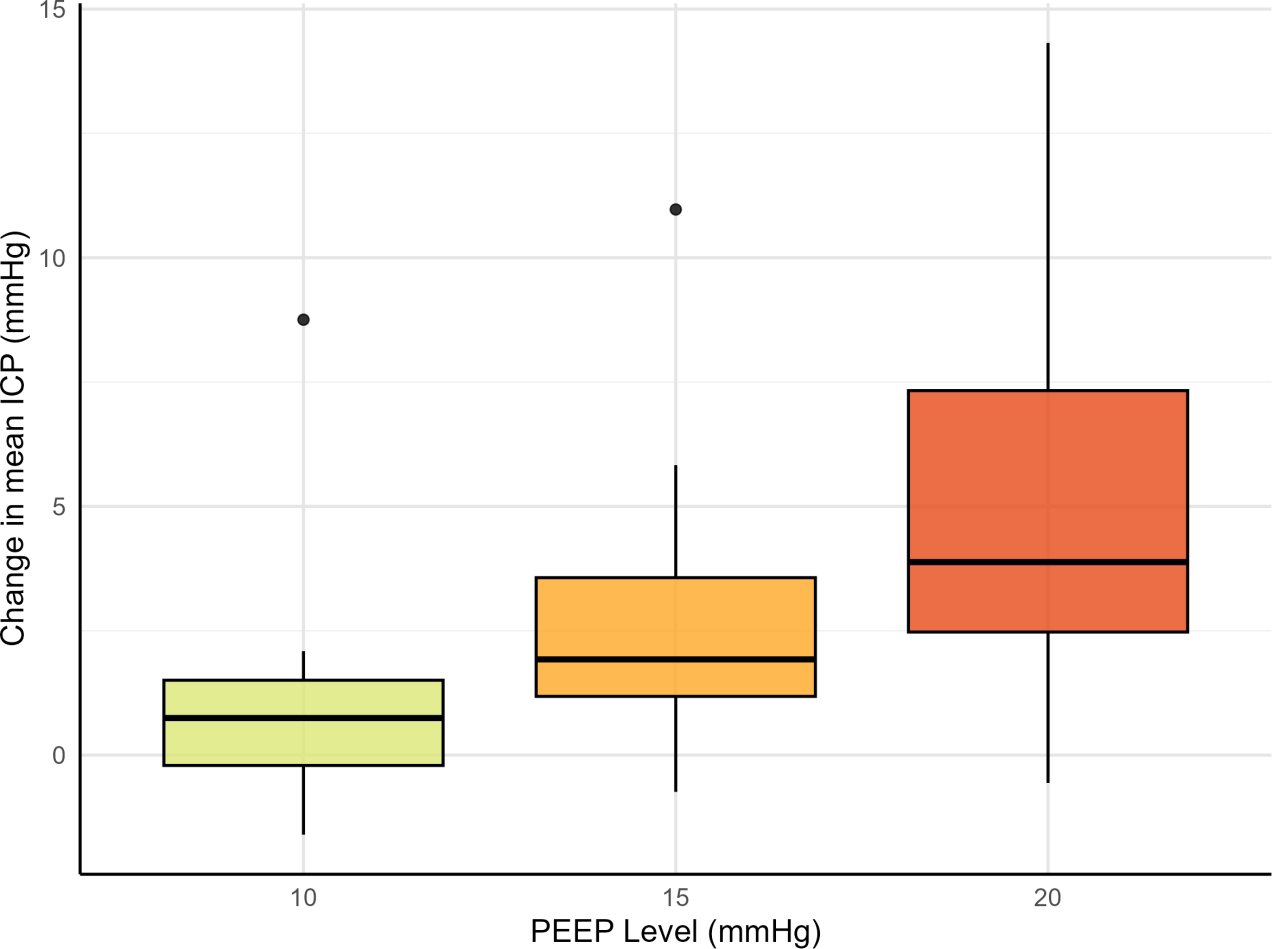
Mean change in ICP with PEEP at 10, 15 and 20 cmH_2_O from baseline PEEP 5 cmH2O. The change in intracranial pressure (ICP) was calculated as the increase from the baseline at positive end-expiratory pressure (PEEP) 5 cmH_2_O. There was a significant association between PEEP levels and change in mean ICP (p < 0.001). The prone and supine data are pooled.

A significant association was observed between PEEP level and change in ICP (p < 0.001), as illustrated in **Figure 3**. Because no differences in ICP response were found between prone and supine positions, data from both positions were pooled.

Relative to baseline at PEEP 5 cmH_2_O, the mean changes in ICP at PEEP levels of 10, 15, and 20 cmH_2_O compared to baseline were 0.9 ± 2.0 mmHg, 2.5 ± 2.4 mmHg, and 5.2 ± 4.3 mmHg, respectively. Summary boxplots of mean change in ICP at each PEEP level in prone and supine are provided in **Supplementary Figures 1A, B**.

### 3.3 Effects of PEEP on PRx

Assumptions of the crossover design were met for PRx, with no significant carryover, sequence, or period effects.

**Figure 4:**
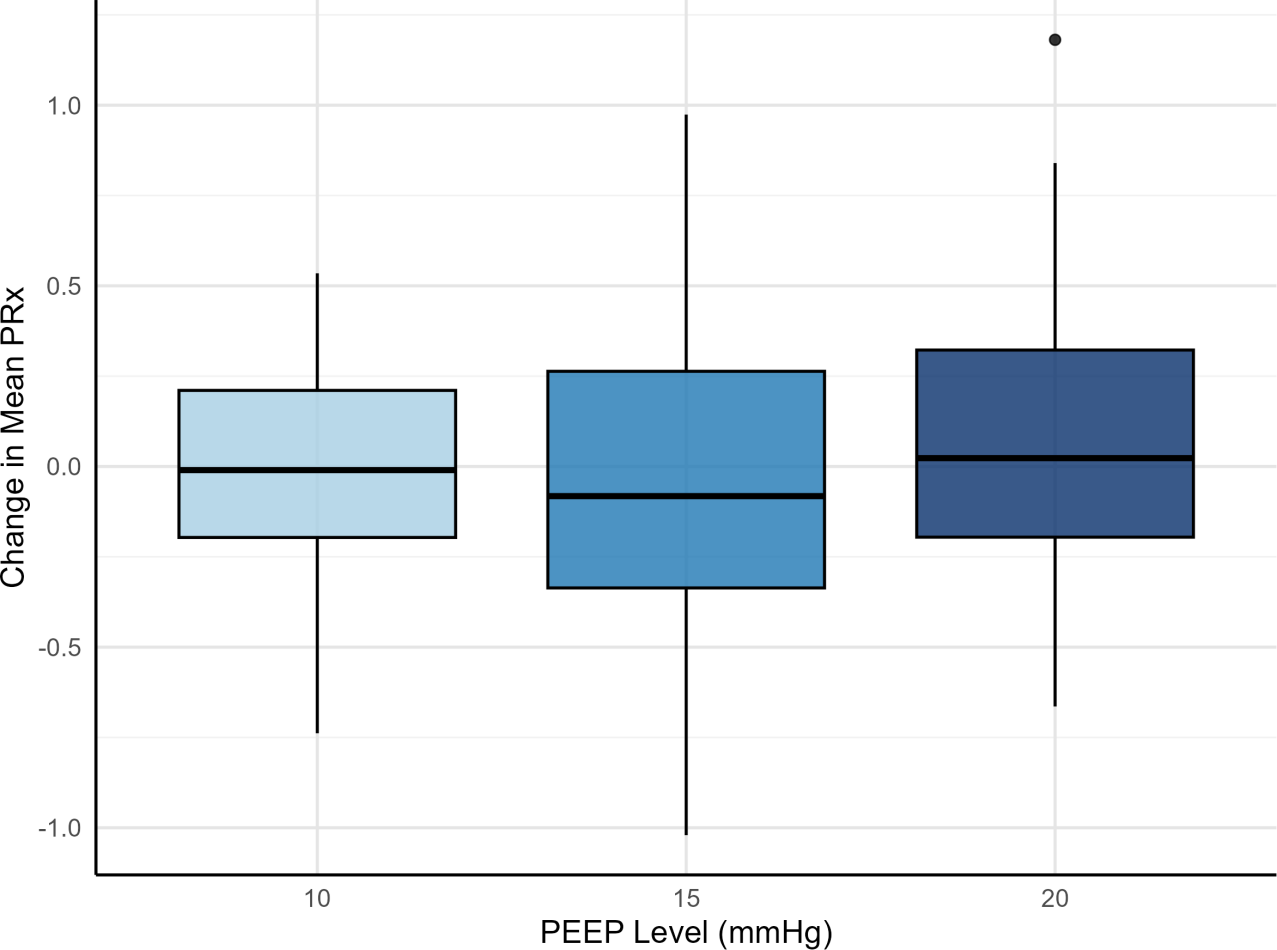
Mean change in pressure reactivity index (PRx) at increasing PEEP levels.Boxplot showing the distribution of the change in PRx from baseline value at PEEP 5 cm H_2_O at PEEP 10, 15, and 20 cmH_2_O. There was no significant association between PEEP and PRx (p = 0.61). Data from prone and supine positions are pooled.

PEEP elevation did not significantly affect mean PRx compared with baseline (p = 0.61) in this study setting where CPP was held above 60 mmHg. Given the absence of a position effect or interaction, data from prone and supine positions were pooled (**Figure 4**). The mean PRx changes were −0.01 ± 0.31, −0.05 ± 0.47, and 0.07 ± 0.47 at PEEP levels of 10, 15, and 20 cmH_2_O, respectively.

### 3.4 Effects of PEEP on brain tissue oxygenation

PbO_2_ tended to increase with PEEP without reaching statistical significance: in prone, median 23 (22–27) to 40 (27–67) mmHg (p = 0.23), and in supine, 21 (15–40) to 52 (35–83) mmHg (p = 0.17) from PEEP 5 to 20 (see **Supplementary Tables 2A, B**).

To adjust for systemic oxygenation, we assessed the PbO_2_/PaO_2_ ratio across PEEP levels separately for prone and supine positions (**Supplementary Tables 3A, B**). The ratio remained relatively stable across PEEP levels in both positions, without a consistent PEEP-dependent trend.

### 3.5 Baseline variables associated with the ICP response

Further exploratory analysis investigated whether selected baseline physiological variables, as presented in **Table 1**, were associated with changes in ICP induced by PEEP. In the linear mixed-effects model, a higher baseline mean respiratory rate (RR) was associated with attenuated ICP response (p = 0.03). Higher baseline mean pulmonary artery pressure (PAP) (p = 0.002) was significantly associated with higher ICP response. No significant associations were found for the other tested baseline variables: ICP, RAP, PaO_2_, PaCO_2_, VTinsp, Pawei, TPPei, TPPee, Ers, El, MPrs, CVP, and CO.

### 3.6 PEEP-responsiveness of the ARDS model

PaO_2_/FiO_2_ ratio and elastance of the ARDS model are shown in **Tables 2A** (prone) and **2B** (supine).

**Table 2:** (A, B) Elastance and oxygenation at PEEP 5–20 cmH_2_O in the ARDS model, prone and supine.

2A					
Variables in prone position	PEEP 5	PEEP 10	PEEP 15	PEEP 20	p
Ers (cmH_2_O/L)	83 (79-94)	79 (73-96)	87 (73-96)	112 (104-121)	**<0.01**
Ecw (cmH_2_O/L)	17 (15-22)	15 (11-18)	19 (13-20)	19 (14-28)	0.33
El (cmH_2_O/L)	67 (56-73)	66 (58-77)	72 (69-73)	95 (89-101)	**<0.01**
PaO_2_/FiO_2_ (mmHg)	182 (162-230)	274 (157-350)	397 (336-462)	341 (261-457)	**0.01**
2B					
Variables in supine position	PEEP 5	PEEP 10	PEEP 15	PEEP 20	p
Ers (cmH_2_O/L)	95 (86-116)	95 (85-134)	96 (83-125)	114 (104-131)	0.35
Ecw (cmH_2_O/L)	17 (12-22)	17 (8-25)	16 (1-20)	17 (13-23)	0.94
El (cmH_2_O/L)	78 (70-97)	81 (65-118)	85 (68-110)	101 (86-110)	0.56
PaO_2_/FiO_2_ (mmHg)	99 (74-175)	198 (122-269)	249 (177-455)	371 (273-440)	**<0.01**

Values are median (IQR). p-values show overall differences between PEEP levels for each variable (one-way ANOVA for normally distributed data; Welch’s ANOVA when variances were unequal; Kruskal–Wallis test for non-normally distributed data). Ers, respiratory system elastance; Ecw, chest wall elastance; El, lung elastance; PaO_2_/FiO_2_, oxygenation index; PEEP, positive end-expiratory pressure; IQR, interquartile range.

Bold values indicate statistically significant differences.

At baseline, the model demonstrated reduced PaO_2_/FiO_2_ and increased elastance compared to healthy lungs (24). PaO_2_/FiO_2_ improved with higher PEEP in both the prone (p = 0.01) and supine (p < 0.01) positions. In supine, PaO_2_/FiO_2_ increased continuously with rising PEEP, while in prone, PaO_2_/FiO_2_ improved up to PEEP 15 cmH_2_O followed by a drop at PEEP 20 cmH_2_O.

In prone, Ers and El increased with PEEP (both p < 0.01), while Ecw was unchanged (p = 0.33); in supine, elastances showed no significant changes.

Comprehensive panels across PEEP levels are provided in **Supplementary Tables 2A, B**, which also show that PaCO_2_ and CPP remained stable across PEEP in both positions. Notably, maintaining stable PaCO_2_ required a significant increase in RR with rising PEEP in both prone (p < 0.01) and supine (p < 0.01) positions.

### 3.7 Comparison of ICP healthy vs ARDS lungs: ICP and respiratory response to PEEP

**Figure 5:**
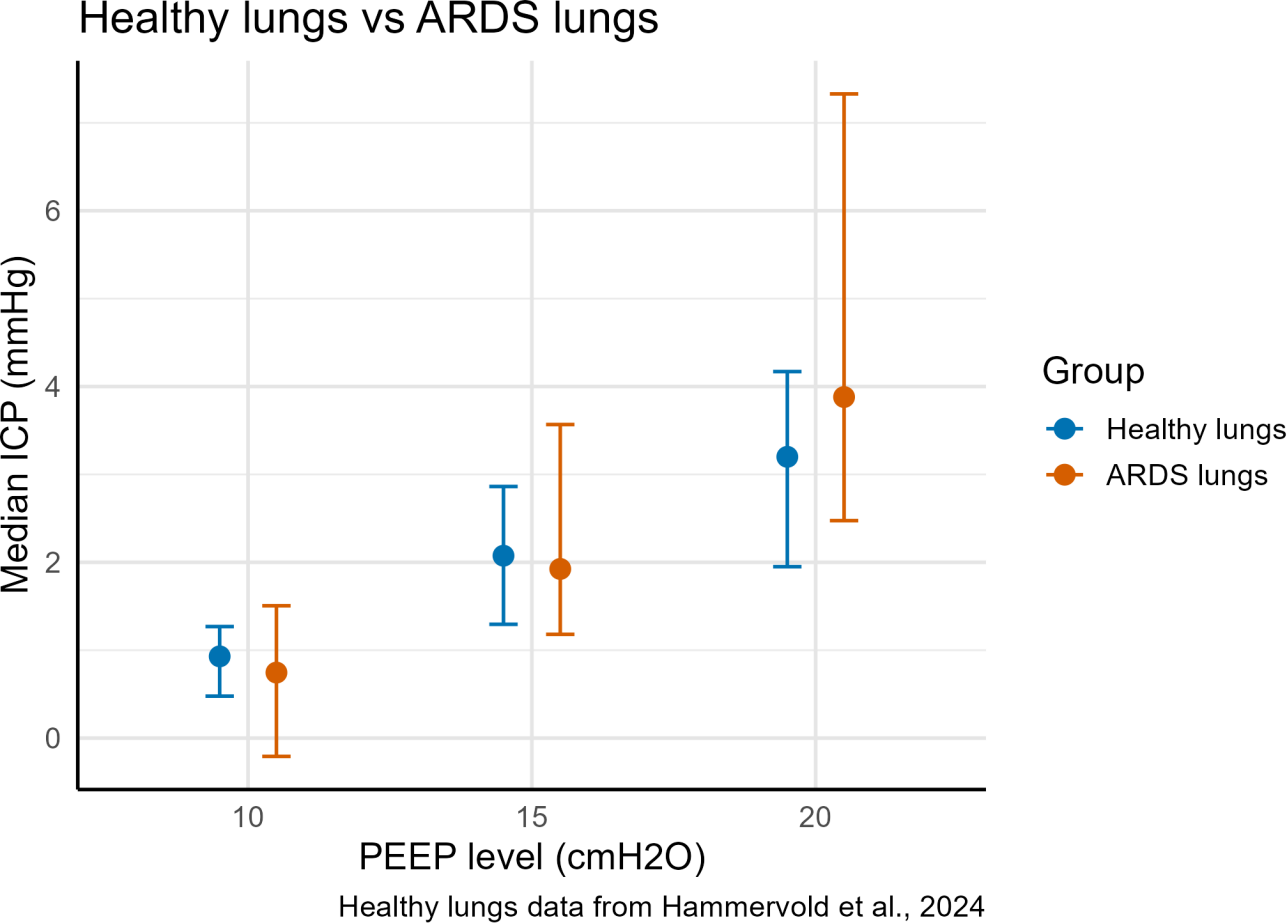
Change in intracranial pressure (ICP) from baseline (PEEP 5) with increasing positive end-expiratory pressure (PEEP) in animals with healthy vs. ARDS lungs Boxplots at PEEP 10, 15, and 20 cmH_2_O). ICP, intracranial pressure; PEEP, positive end-expiratory pressure; ARDS, acute respiratory distress syndrome.

We compared the present ARDS data with our previous study of animals with healthy lungs studied under the same crossover protocol (Hammervold et al., 2024). **Figure 5** shows the distribution at PEEP 10, 15, and 20 cmH_2_O. Medians and IQRs are summarized descriptively in **Supplementary Table 4**. No clear differences in the pattern of ICP response between the healthy and ARDS groups were observed across PEEP levels.

## 4 Discussion

In this porcine ARDS model induced by saline lung lavage followed by injurious ventilation, stepwise increases in PEEP resulted in a rise in intracranial pressure (ICP) that was not modified by body position. The cerebrovascular reactivity (PRx) remained unchanged across PEEP levels.

The magnitude of the ICP rise with increasing PEEP in this ARDS model was similar to that observed previously in healthy lungs, despite lower compliance, higher end-inspiratory respiratory-system and transpulmonary pressures, and a lower PaO_2_/FiO_2_ ratio (Hammervold et al., 2024). PaCO_2_ was slightly higher in the ARDS model, without a greater influence on the ICP response. CPP was similar in the two studies. The similar ICP response therefore suggests that the effect of PEEP on ICP is likely not primarily determined by lung compliance alone.

A previous experimental work in a porcine lung injury model reveals small, directionally consistent increases in ICP with higher PEEP, consistent with our data (Chen et al., 2021). A similar minor response in ICP with increasing PEEP was also observed in a feline model with pulmonary dysfunction (Aidinis et al., 1976). A separate porcine ARDS study described more pronounced ICP rises while using airway pressure release ventilation (APRV) and lung-protective ventilation (LPV), although exact PEEP levels were not specified, limiting comparability (Davies et al., 2015).

In patient cohorts with acute brain injury (ABI), higher PEEP is associated with small ICP increases (Borsellino et al., 2016; Boone et al., 2017; Giardina et al., 2023; Zunino et al., 2024). Xiu et al. noted that PEEP >10 cmH_2_O in mechanically ventilated acute neurological injury patients without ARDS was linked to modest ICP increases alongside improved oxygenation and respiratory mechanics, whereas low–moderate PEEP (5–10 cmH_2_O) had minimal impact on these parameters (Xiu, 2025). In our previous clinical study, BrainVent, we found that while most patients with ABI and no ARDS showed a significant but clinically non-relevant increase in ICP, nearly one-quarter of the patients experienced a rise in ICP to above 20 mmHg during the increases in PEEP, necessitating its interruption (Beqiri et al., 2023). The increase in ICP was not related to respiratory compliance but rather associated with lower brain compliance at the baseline. A recent systematic review on lung-brain interactions reported that while PEEP can induce a raise in ICP, the magnitude and clinical relevance of this increase varies across different studies and patient populations (Mascia et al., 2025).

Chen et al. reported a chest wall compliance-dependent response in their porcine study. The reduced compliance was a result of chest-wall strapping, rather than the reduced lung compliance caused by the ARDS lung injury model (Chen et al., 2021). A clinical study with SAH patients also found that lower chest wall compliance increased the ICP response to PEEP (Chen et al., 2018). In our study, lung compliance decreased as expected in ARDS, while chest wall compliance remained unchanged compared to healthy lungs. This may explain why the ICP response to PEEP was not more pronounced in the ARDS model than in healthy lungs. The ICP response to increasing PEEP was similar in prone and supine positions and did not differ from what we have previously observed in healthy lungs (Hammervold et al., 2024). At PEEP 20 cmH_2_O in prone, oxygenation declined and elastance increased, suggesting that this level may have approached the limit of net recruitment. At baseline, the supine position showed a slightly negative end-expiratory transpulmonary pressure (TPPee) and a modestly higher respiratory system driving pressure. A negative TPPee derived from esophageal pressure may indicate derecruitment or compression in dependent-to-mid lung regions adjacent to the esophagus, rather than collapse of the entire lung (Yoshida et al., 2018; Ball et al., 2024).

Moreover, values close to zero (approximately −2 to +2 cmH_2_O) have been considered within a physiologically acceptable or protective range in studies using transpulmonary pressure–guided ventilation (Talmor et al., 2008; Sarge et al., 2021).

Driving pressure reflects the mechanical properties of the entire respiratory system and may increase with higher pleural or chest-wall pressure without implying greater intrinsic lung stress. Consistent with this, mechanical power, which depends on tidal volume, respiratory rate, and absolute pressure levels, remained similar between positions (Gattinoni et al., 2016b). We therefore conclude that in our experimental setting the respiratory mechanics in prone and supine were overall similar.

Although negative end-expiratory transpulmonary pressure may theoretically promote pulmonary edema, this mechanism is mainly associated with large negative pleural pressure swings during spontaneous breathing (Carteaux et al., 2021). In our controlled mechanical ventilation model, TPPee was slightly negative and close to zero and the baseline exposure time limited, which was likely insufficient to induce measurable changes in global extravascular lung water (EVLW).

High airway pressures and lung overdistension can increase pulmonary microvascular permeability and promote pulmonary edema formation (Dreyfuss and Saumon, 1998). In our study, at the highest PEEP level, EVLW increased in prone but remained stable in supine (**Supplementary Tables 2A, B**), indicating position-dependent effects at high PEEP.

In prone, Ers and El increased and PaO_2_/FiO_2_ declined from PEEP 15 to 20, suggesting that this level may have shifted the balance from recruitment toward overdistension (Crotti et al., 2001; Gattinoni et al., 2016a). Overdistension of non-dependent lung regions may also alter pulmonary perfusion. Compression of alveolar capillaries in overdistended areas can redistribute blood flow toward less distended and better-ventilated lung regions, which may help preserve overall ventilation–perfusion matching despite reduced ventilation in the overinflated zones (Gattinoni et al., 2013).

Systemic hemodynamics remained stable across PEEP levels in both prone and supine positions, with no clinically relevant changes in mean arterial pressure, cardiac output, or central venous pressure. This suggests that the observed respiratory and cerebral effects were not primarily driven by major circulatory changes. These findings indicate that the physiological effects of high PEEP differed between positions despite similar systemic hemodynamic stability, underscoring the importance of lung mechanical responses when interpreting position-dependent effects.

In surgical patients without ARDS, Taj et al. performed transcranial Doppler (TCD)-based ICP (nICP) and pulsatility index (PI) measurements which were unchanged in prone vs supine and remained unchanged with PEEP 5 cmH_2_O (Taj et al., 2025). By contrast, Robba et al. reported higher TCD-derived ICP estimates in prone with a further rise at PEEP 8 cmH_2_O in patients undergoing spine surgery (Robba et al., 2017). Both studies were performed on patients without ARDS, limiting direct comparison to ARDS physiology. Differences in patient characteristics and PEEP level (5 vs 8 cmH_2_O) likely contributed to the discrepant findings. In severe ARDS with brain injury, Elmaleh et al. observed no reduction in TCD surrogates of cerebral blood flow during prone sessions, supporting feasibility of prone position in those patients provided close monitoring is applied (Elmaleh et al., 2024).

PaCO_2_ was kept stable across PEEP levels in our model. In other study settings where position might affect PaCO_2_, the ICP response might be different to our result. Given the well-established benefits of prone positioning for oxygenation in ARDS (Gattinoni et al., 2013; Guérin et al., 2020), these data support its continued use when cerebral physiology is monitored and remains stable. These findings suggest that prone ventilation may be feasible when physiological targets such as adequate MAP/CPP, normocapnia, normovolemia, and avoidance of excessive lung inflation are actively maintained, rather than relying on monitoring alone, consistent with proposed protocols for prone ventilation in neurologically ill patients (Wright et al., 2021).

To our knowledge, this is the first porcine ARDS study to examine PRx responses across graded PEEP. PRx is increasingly recognized as a robust surrogate marker for cerebrovascular autoregulation in traumatic brain injury (TBI) patients, and is used to guide individualized management in TBI (Tsigaras et al., 2023; Park et al., 2024). The validity of PRx in animal models, including pigs, has been confirmed in several experimental settings (Liu et al., 2020; Klein et al., 2024).

PRx remained unchanged across PEEP levels, indicating preserved dynamic autoregulation despite lung injury. This extends our prior observations in healthy pigs (Hammervold et al., 2024) and aligns with studies in brain-injured patients where adjustments in ventilatory pressures, including PEEP, were not associated with deterioration of PRx under controlled conditions (Beqiri et al., 2023; Tsigaras et al., 2023).

In our stepwise moderated rise in PEEP protocol, ICP did not rise to the point of decreasing CPP below the lower limit of autoregulation and PaCO_2_ was maintained constant. CPP and PaCO_2_ are two important modulators of CA. Given cerebrovascular reactivity did not change across PEEP values, we can conclude that increasing PEEP alone did not induce vascular paralysis either.

In our model, PbO_2_ increased with higher PEEP while the PbO_2_/PaO_2_ ratio remained stable, consistent with a systemic oxygenation–driven effect rather than a direct cerebrovascular effect of PEEP. This aligns with TBI literature, indicating that PbO_2_ is sensitive to systemic oxygen delivery (Jeffcote et al., 2024). In TBI cohorts, lower PaO_2_/FiO_2_ independently predicts cerebral hypoxia and mortality, underscoring the lung–brain link and supporting cautious use of PEEP to improve gas exchange when PaCO_2_ and CPP are controlled (Robba et al., 2020a). Although brain tissue oxygenation increased with higher PEEP, improved oxygenation does not necessarily indicate preserved cerebral microcirculation or metabolism. ARDS is associated with systemic inflammation, endothelial dysfunction, and blood–brain barrier disruption that may affect the brain independently of macrocirculatory variables (Ziaka and Exadaktylos, 2022). Experimental studies further suggest that cerebral oxygenation may improve despite persistent microcirculatory or metabolic disturbances (Taccone et al., 2022). Because such parameters were not measured in the present study, these mechanisms cannot be excluded.

Mechanical ventilation and lung injury may influence the brain through mechanisms beyond systemic hemodynamics. Lung injury can trigger systemic inflammatory responses with potential effects on the blood–brain barrier, cerebral edema, intracranial pressure, and cerebrovascular autoregulation (Mascia et al., 2025). These mechanisms were not assessed in the present study. However, ICP and PRx responses were not different in this lung injury model compared with healthy lungs.

The ICP response to PEEP was similar in healthy and ARDS lungs. Because the studies were conducted at different time periods under potentially different conditions, no formal between-study statistical testing was performed. However, baseline physiological characteristics were comparable in terms of age, weight, cerebral variables (including ICP and CPP), and hemodynamic status, while respiratory differences were consistent with the presence of ARDS in the current study compared with the previous healthy-lung study. These findings support a descriptive comparison of ICP response patterns between the two models.

Increasing PEEP improved oxygenation in both positions, consistent with recruitment and reduced shunt (Chiumello et al., 2008). At the same time, respiratory system and lung elastance increased at higher PEEP levels—particularly in the prone position—compatible with reduced compliance or operation on the upper portion of the pressure–volume curve despite protocol-driven reductions in tidal volume (Crotti et al., 2001; Gattinoni et al., 2016b). This pattern is consistent with the coexistence of recruitment and overdistension in heterogeneous lung injury (Chiumello et al., 2008). Importantly, the increase in intracranial pressure was similar in prone and supine positions despite these differences in lung mechanics, suggesting that ICP elevation was not restricted to conditions compatible with hyperinflation (Robba et al., 2020b). In our analysis, higher baseline mean PAP was associated with a larger ICP increase during PEEP escalation, whereas a higher respiratory rate was associated with a smaller increase. The PAP finding is physiologically plausible: greater pulmonary vascular load may predispose to larger intrathoracic pressure swings and venous congestion during positive pressure, facilitating transmission of pressure to the cranial compartment. Consistent with this, baseline PAP was higher in ARDS than in healthy lungs, and only at higher baseline PAP, which only occurred in ARDS lungs, did we observe an association between PEEP and the ICP response.

Although the specific influence of baseline PAP on the PEEP–ICP relationship has not been studied directly, experimental work supports bidirectional lung–brain cross-talk. For example, in pigs, acute intracranial hypertension increased extravascular lung water and CT lung density in both healthy and ARDS lungs, supporting the plausibility that pulmonary vascular load conditions intracranial responses to ventilatory pressure (Heuer et al., 2011). We did not see a similar association with CVP as we did in PAP. CVP was similar in ARDS and healthy lungs.

The mechanism underlying the RR finding remains uncertain; potential explanations include lower tidal volumes at higher RR (limiting airway/plateau pressures) and slightly higher PaCO_2_ prompting a compensatory increase in RR. Alternatively, small transient rises in PaCO_2_ during PEEP elevation could have influenced ICP, although these variations were promptly corrected through ventilatory adjustments. In addition, a higher RR may have increased relative mechanical power, which could also modulate the ICP response, although this was not quantified in the present analysis. Given the sample size, subgroup analyses were not feasible, and we are not aware of prior reports demonstrating the same pattern.

### 4.1 Strengths and limitations

The main strength of this study is the use of a controlled experimental ARDS model with multimodal monitoring of respiratory, hemodynamic, and cerebral parameters. This design allowed for precise control of variables, including strict PaCO_2_ regulation, thereby minimizing confounding effects and enabling the isolated assessment of PEEP on ICP and cerebral autoregulation in both prone and supine positions. Another important strength is the use of the same experimental protocol as in our previous healthy-lung study, allowing for direct comparison and interpretation of differences between healthy and injured lungs.

However, several limitations should be acknowledged. As with any animal model, anatomical and physiological differences may limit the direct translation of our findings to clinical practice.

The PEEP levels were applied in a fixed, non-randomized order without recruitment maneuvers between steps. This approach was chosen to maintain stable hemodynamics and minimize potential confounding effects on ICP and PRx but may have influenced lung mechanics and limited the assessment of isolated PEEP effects. Furthermore, the short experimental duration precluded evaluation of longer-term effects of PEEP on cerebral and respiratory parameters.

Although PaCO_2_ was tightly controlled in our study, different physiological conditions—such as hypercapnia, hypotension with reduced CPP, impaired cerebrovascular autoregulation, or greater chest wall stiffness—might yield different responses. In clinical ARDS management, permissive hypercapnia is often accepted to maintain lung-protective ventilation, whereas our protocol prioritized normal PaCO_2_ alongside low ICP and controlled airway pressures. These combined targets may be challenging to achieve simultaneously in real-world settings.

Fluid administration and vasopressor therapy were protocolized to maintain cerebral perfusion pressure within the target range. However, detailed stage-specific cumulative fluid balance and norepinephrine doses were not systematically recorded for each PEEP level.

Cerebral metabolism, inflammatory mediators, and neuronal electrical activity (EEG) were not measured; therefore, microcirculatory or metabolic disturbances cannot be excluded despite stable ICP, PRx, and brain tissue oxygenation. Imaging-based assessment of alveolar recruitment or hyperinflation (e.g., CT or electrical impedance tomography) was not performed; therefore, the relative contributions of recruitment and overdistension could not be measured.

## 5 Conclusion

This study indicates that moderate PEEP escalation, under controlled hemodynamic and ventilatory conditions, produces only a small increase in ICP without compromising PRx or PbO_2_, in animals with ARDS and healthy brain. Future studies should address whether these relationships persist in the setting of intracranial hypertension, where autoregulatory capacity and intracranial compliance may be impaired. This will better inform ventilatory strategies when both lung and brain physiology are at risk.

## Data Availability

The raw data supporting the conclusions of this article will be made available by the authors, without undue reservation.

## Glossary

ABP: Arterial blood pressure
ABI: Acute brain injury
ARDS: Acute respiratory distress syndrome
AMP: Intracranial pulse amplitude
AuxPressure: Esophageal pressure (auxiliary pressure channel in ICM+)
CA: Cerebral autoregulation
CO: Cardiac output
CPP: Cerebral perfusion pressure
CVP: Central venous pressure
DP: Driving pressure
Ecw: Chest wall elastance
El: Lung elastance
Ers: Respiratory system elastance
EVLW: Extravascular lung water
FiO₂: Fraction of inspired oxygen
HR: Heart rate
ICP: Intracranial pressure
ICM+: Intensive Care Monitoring Plus software
IQR: Interquartile range
LPV: Lung-protective ventilation
MAP: Mean arterial pressure
MPlDep: Mechanical power in dependent lung region
MPlnonDep: Mechanical power in non-dependent lung region
MPrs: Mechanical power of the respiratory system
PaCO₂: Arterial partial pressure of carbon dioxide
PaO₂: Arterial partial pressure of oxygen
PAP: Pulmonary artery pressure
PbO₂: Brain tissue oxygen tension
PEEP: Positive end-expiratory pressure
Pesee: End-expiratory esophageal pressure
Pesei: End-inspiratory esophageal pressure
Ppeakcw: Peak inspiratory pressure of the chest wall
Ppeakl: Peak inspiratory pressure of the lung
Ppeakrs: Peak inspiratory pressure of the respiratory system
PRx: Pressure reactivity index
RAP: Compensatory reserve index
RR: Respiratory rate
SD: Standard deviation
SpO₂: Peripheral oxygen saturation
SVV: Stroke volume variation
TPP: Transpulmonary pressure
TPPelast: Elastance-derived transpulmonary pressure
TPPee: End-expiratory transpulmonary pressure
TPPei: End-inspiratory transpulmonary pressure
TBI: Traumatic brain injury
VTinsp: Inspiratory tidal volume
