# Supplementary material for: Positive end-expiratory pressure increases intracranial pressure but does not affect PRx, regardless of body position, in a porcine ARDS model
Source: Front Physiol. 2026 Mar 25;17:1792273. doi: 10.3389/fphys.2026.1792273 (PMC13056657; doi:10.3389/fphys.2026.1792273)
Supplement: Supplementary file 1 [file DataSheet1.docx]

**Supplementary Material**

**Supplementary Figure S1.** Mean intracranial pressure at increasing levels of positive end-expiratory pressure.

Mean intracranial pressure (ICP) is shown at different levels of positive end-expiratory pressure (PEEP) in (a) prone and (b) supine positions. Values are pooled across sequence.


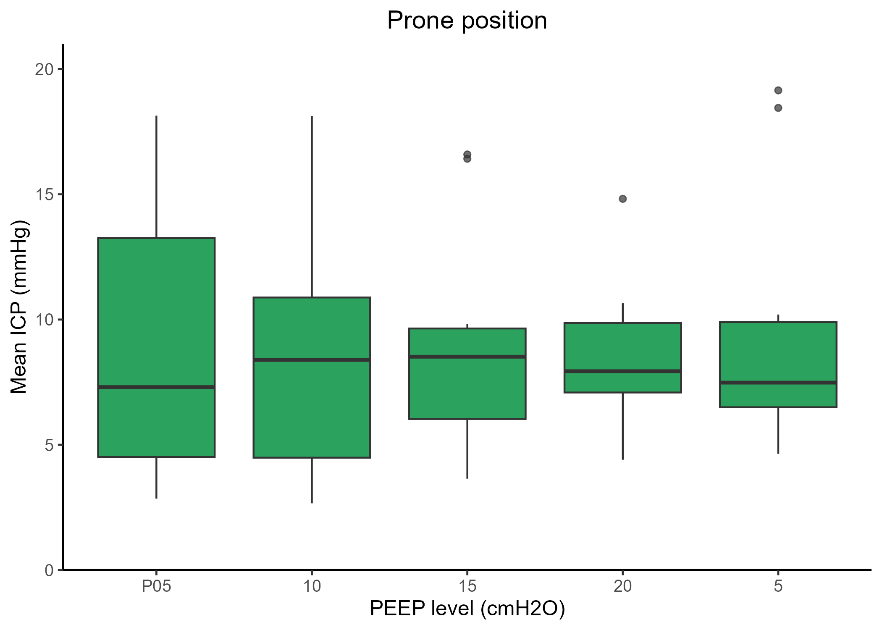


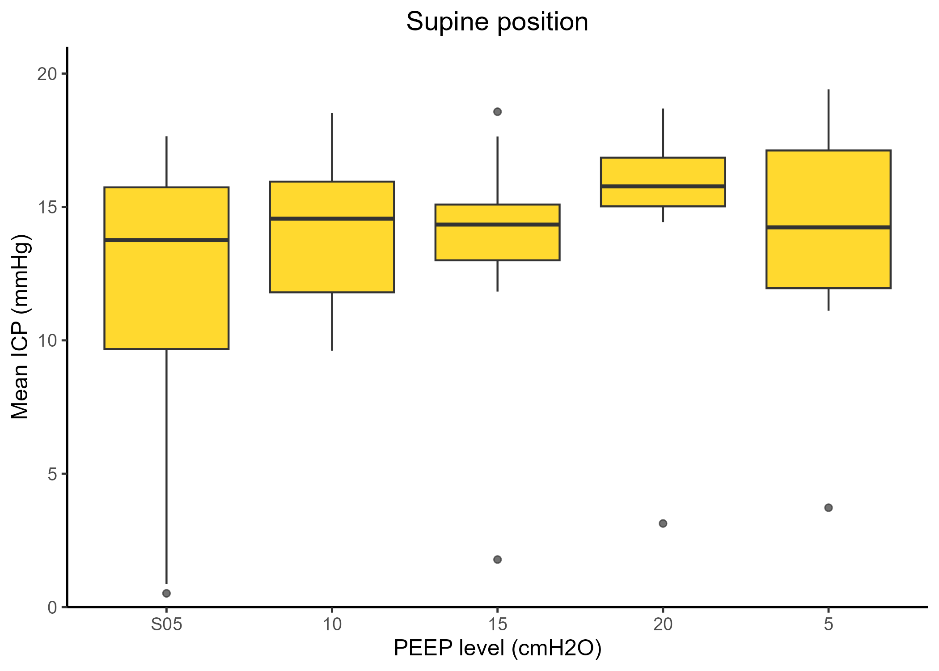


**Supplementary Table S1.** Calculation formulas for derived respiratory mechanics variables

All variables were obtained as described in the Methods section; derived variables were calculated using the formulas shown below.

|  |  |  |
| --- | --- | --- |
| **Variable** | **Formula / Definition** | **Unit** |
| Ppeakl (peak transpulmonary pressure) | Ppeakrs – Ppeakcw | cmH₂O |
| Ers (respiratory system elastance) | (Pawei – PEEPtot) / VT | cmH₂O/L |
| Ecw (chest wall elastance) | (Pesei – Pesee) / VT | cmH₂O/L |
| El (lung elastance) | Ers – Ecw | cmH₂O/L |
| DP (driving pressure) | Pawei – PEEPtot | cmH₂O |
| TPPee (end-exp. transpulmonary pressure) | PEEPtot – Pesee | cmH₂O |
| TPPei (end-insp. transpulmonary pressure) | Pawei – Pesei | cmH₂O |
| TPPelast (elastance-derived transpulmonary pressure) | Pawei × (El / Ers) | cmH₂O |
| MPrs (mechanical power, respiratory system) | 0.098 × VT × RR × (Ppeakrs – (Pawei – Pesee)/2) | J/min |
| MPlDep (mechanical power, dependent lung) | 0.098 × VT × RR × (Ppeakl – (TPPei – TPPee)/2) | J/min |
| MPlnonDep (mechanical power, non-dep. lung) | 0.098 × VT × RR × (Ppeakl – (TPPelast – TPPee)/2) | J/min |

**Supplementary Table S2A.** Cerebral, respiratory, and hemodynamic variables at different PEEP levels in prone position.

P-values indicate whether there are statistically significant differences between PEEP levels for each variable.

|  |  |  |  |  |  |
| --- | --- | --- | --- | --- | --- |
| Variable | S5 | S10 | S15 | S20 | p |
| Cerebral Variables |  |  |  |  |  |
| ICP (mmHg) | 15 (11-18) | 15 (12-17) | 15 (14-19) | 17 (15-21) | 0.27 |
| PRx | (-)0.064 ± 0.240 | -0.156 ± 0.254 | -0.087 ± 0.311 | -0.005 ± 0.301 | 0.61 |
| RAP | 0.131 ± 0.153 | 0.081 ± 0.231 | 0.105 ± 0.179 | 0.027 ± 0.169 | 0.56 |
| CPP (mmHg) | 73 ± 8 | 75 ± 12 | 72 ± 7 | 71 ± 7 | 0.80 |
| PbO2 (mmHg) | 21 (15-40) | 27 (14-45) | 46 (32-71) | 52 (35-83) | 0.17 |
| Respiratory Variables |  |  |  |  |  |
| PaCO2 (mmHg) | 44 ± 6 | 44 ± 6 | 45 ± 10 | 54 ± 19 | 0.43 |
| PaO2 (mmHg) | 74 (68-98) | 99 (98-195) | 150 (113-183) | 173 (128-319) | **<0.01** |
| PaO2/FiO2 (mmHg) | 99 (74-175) | 198 (122-269) | 249 (177-455) | 371 (273-440) | **<0.01** |
| VTinsp (ml) | 195 ± 23 | 188 ± 30 | 173 ± 32 | 146 ± 29 | **<0.01** |
| RR (breaths/min) | 36 (32-39) | 36 (32-38) | 40 (34-48) | 47 (46-47) | **<0.01** |
| Ppeakrs (cmH2O) | 36.8 (30.6-40.7) | 39 (34-42) | 43 (40-45) | 45 (44-47) | **<0.01** |
| Ppeakcw (cmH2O) | 12.2 (11.2-13.9) | 14.2 (12.5-16.8) | 15.2 (12.4-16.3) | 16 (14-18) | 0.07 |
| Ppeakl (cmH2O) | 23.3 (19.3-28.1) | 22.1 (16.2-29.6) | 28.2 (23.2-31.8) | 29 (26-34) | 0.14 |
| Pawei (cmH2O) (PawInsp) | 24.9 (21.7-28.7) | 30.7 (25.3-33.8) | 33.5 (30.5-36.3) | 38.4 (35.9-38.8) | **<0.01** |
| Pawee (cmH2O) (PawExp) | 5.6 (5.2-5.9) | 10.6 (10.3-10.8) | 15.7 (15.6-16.1) | 20.8 (20.5-21.5) | **<0.01** |
| TPPei (cmH2O) (PlInsp) | 16.5 (13.7-18.9) | 19.6 (12.7-24.4) | 21.4 (19.2-25.3) | 23.7 (21.4-26.7) | **<0.01** |
| TPPee (cmH2O) (Plexp) | (-)0.9 ((-)1.7-(-)0.3) | 1.9 ((-)1-3.8) | 6.3 (5.2-7.8) | 9.8 (9.3-11.8) | **<0.01** |
| TPPelast (cmH2O) | 22.1 (17.1-23.6) | 25.0 (20.6-29.5) | 29.8 (27.2-30.6) | 30.8 (29.4-34.1) | **<0.01** |
| Pesei (cmH2O) (PesoInsp) | 9.9 ± 2.7 | 11.5 ± 3.2 | 12.0 ± 2.9 | 13.5 ± 3.3 | 0.05 |
| Pesee (cmH2O) (PesoExp) | 6.5 ± 2.3 | 8.7 ± 2.6 | 9.5 ± 2.6 | 10.9 ± 2.6 | **<0.01** |
| Ers (cmH2O/l) | 95 (86-116) | 95 (85-134) | 96 (83-125) | 114 (104-131) | 0.35 |
| Ecw (cmH2O/l) | 17 (12-22) | 17 (8-25) | 16 (1-20) | 17 (13-23) | 0.94 |
| El (cmH2O/l) | 78 (70-97) | 81(65-118) | 85 (68-110) | 101 (86-110) | 0.56 |
| DP (cmH₂O) | 20.08 ± 4.48 | 19.87 ± 5.41 | 17.58 ± 3.42 | 16.74 ± 2.91 | **<0.01** |
| MPrs (J/min) | 17 (14-19) | 17 (16-18) | 19 (19-24) | 22 (21-26) | **0.02** |
| MPlDep (J/min) | 10 (7-12) | 9 (7-12) | 13 (10-16) | 15 (12-16) | 0.13 |
| MPlnonDep (J/min) | 9 (6-11) | 7 (5-11) | 10 (7-14) | 12 (10-15) | 0.27 |
| Hemodynamic Variables |  |  |  |  |  |
| ABP mean (mmHg) | 86 ± 6 | 90 ± 11 | 89 ± 7 | 90 ± 7 | 0.63 |
| CVP mean (mmHg) | 13 (11-15) | 13 (11-14) | 13 (12-15) | 14 (14-16) | 0.16 |
| PAP mean (mmHg) | 35 (30-42) | 33 (28-41) | 35.3 (29.4-42.2) | 42 (33-43) | 0.46 |
| CO (L/min) | 3.2 (2.9-3.3) | 3.4 (2.9-5.0) | 3.4 (2.9-5.0) | 3.3 (2.7-5.0) | 0.72 |
| EVLW (mL) | 503 (394-616) | Not measured | Not measured | 493 (430-604) | 0.86 |
| SVV (%) | 11 ± 4 | Not measured | Not measured | 16 ± 4 | 0.79 |
| Temperature (°C) | 38.6 ± 0.3 | 38.5 ± 0.3 | 38.7 ± 0.3 | 38.8 ± 0.2 | 0.09 |
| **Supplementary Table S2B.** Cerebral, respiratory, and hemodynamic variables at different PEEP levels in supine position.  P-values indicate whether there are statistically significant differences between PEEP levels for each variable. | | | | | |
|  |  |  |  |  |  |
|  |  |  |  |  |  |
| Variable | P5 | P10 | P15 | P20 | p |
| Cerebral Variables |  |  |  |  |  |
| ICP (mmHg) | 7 (5-13) | 8 (5-13) | 9 (6-17) | 10 (8-22) | 0.33 |
| PRx | (-)0.113 ± 0.194 | (-)0.045 ± 0.224 | (-)0.157 ± 0.373 | (-)0.041±0.403 | 0.76 |
| RAP | (-)0.021 ± 0.148 | (-)0.020 ± 0.196 | 0.042 ± 0.122 | 0.057 ± 0.102 | 0.42 |
| CPP (mmHg) | 80 ± 14 | 77 ± 13 | 73 ± 11 | 70 ± 9 | 0.15 |
| PbO2 (mmHg) | 23 (22-27) | 30(26-33) | 34 (22-46) | 40 (27-67) | 0.23 |
| Respiratory Variables |  |  |  |  |  |
| PaCO2 (mmHg) | 46 ± 7 | 43 ± 6 | 42 ± 5 | 52 ± 15 | 0.11 |
| PaO₂ (mmHg) | 99 (85-167) | 134 (112-164) | 172 (151-187) | 190 (133-245) | 0.08 |
| PaO₂/FiO₂ | 182 (162-230) | 274 (157-350) | 397 (336-462) | 341 (261-457) | **0.01** |
| VTinsp (ml) | 204 ± 36 | 198 ± 25 | 182 ± 31 | 138 ± 19 | **<0.01** |
| RR (breaths/min) | 35 (32-37) | 36 (34-39) | 40 (37-46) | 48 (44-50) | **<0.01** |
| Ppeakrs (cmH2O) | 30.7 (28.5-34.1) | 34.7 (32.5-37.7) | 40.8 (36.6-43.7) | 43.6 (41.6-44.4) | **<0.01** |
| Ppeakcw (cmH2O) | 11.0 (9.8-13.2) | 12.6 (10.9-13.7) | 15.6 (13.2-17) | 16.8 (13.3-17.9) | **<0.01** |
| Ppeakl (cmH2O) | 20.8 (17.4-22.1) | 22.3 (20.1-24.5) | 24.7 (23.4-30.4) | 27.6 (26.9-29) | **<0.01** |
| Pawei (cmH2O) (PawInsp) | 22.1 (19.7-24.3) | 25.6 (24.9-29.1) | 32.5 (29.6-34.7) | 36.3 (34.9-37.5) | **<0.01** |
| Pawee (cmH2O) (PawExp) | 5.2 (5-5.7) | 10.6 (10.3-11) | 16.2 (15.6-16.8) | 20.5 (20.3-20.8) | **<0.01** |
| TPPei (cmH2O) (PlInsp) | 13.2 (11.8-15.4) | 16.7 (14.5-18.7) | 20.0 (19.1-21.2) | 23.4 (21.8-24.5) | **<0.01** |
| TPPee (cmH2O) (Plexp) | (-)0.1 ((-)0.6-0.5) | 3.6 (3.1-4.2) | 6.7 (5.6-7.8) | 9.4 (9-11.8) | **<0.01** |
| TPPelast (cmH2O) | 16.7 (15.6-19.1) | 21.7 (20.1-23.5) | 25.7 (24.7-27.0) | 30.4 (27.7-31.9) | **<0.01** |
| Pesei (cmH₂O) | 8.8 ± 2.7 | 10.2 ± 1.3 | 12.7 ± 2.0 | 13.3 ± 2.9 | **<0.01** |
| Pesee (cmH₂O) | 4 .8 ± 2.7. | 7.3 ± 1.7 | 9.5 ± 1.3 | 10.5 ± 1.9 | **<0.01** |
| Ers (cmH₂O/l) | 83 (79-94) | 79 (73-96) | 87 (73-96) | 112 (104-121) | **<0.01** |
| Ecw (cmH₂O/l) | 17 (15-22) | 15 (11-18) | 19 (13-20) | 19 (14-28) | 0.33 |
| El (cmH₂O/l) | 67 (56-73) | 66 (58-77) | 72 (69-73) | 95 (89-101) | **<0.01** |
| DP (cmH₂O) | 17.34 ± 3.41 | 16.16 ± 3.20 | 15.92 ± 3.37 | 15.84 ± 1.83 | **0.23** |
| MPrs (J/min) | 14 (11-19) | 16 (15-23) | 21 (18-30) | 23 (20-26) | 0.06 |
| MPlDep (J/min) | 8 (6-11) | 10 (9-13) | 12 (11-20) | 14 (12-15) | 0.06 |
| MPlnonDep (J/min) | 7 (5-11) | 8 (7-12) | 10 (9-17) | 12 (9-12) | 0.15 |
| Hemodynamic Variables |  |  |  |  |  |
| ABP mean (mmHg) | 89 ± 14 | 87 ± 12 | 84 ± 11 | 84 ± 10 | 0.634 |
| CVP mean (mmHg) | 10 (7-16) | 10 (7-13) | 12 (9-14) | 12 (9-12) | 0.86 |
| PAP mean (mmHg) | 30 (26-34) | 31 (29-33) | 33 (32-34) | 38 (35-39) | **<0.01** |
| CO (L/min) | 2.9 (2.7-3.7) | 3.3 (3.1-3.9) | 3.3 (3.1-3.9) | 3.1 (2.9-3.5) | 0.47 |
| EVLW (mL) | 432 (326-519) | Not measured | Not measured | 679 (516-869) | **0.04** |
| SVV (%) | 12 ± 6 | Not measured | Not measured | 12 ± 3 | 0.98 |
| Temperature (°C) | 38.8 ± 0.4 | 39.0 ± 0.4 | 39.1 ± 0.4 | 38.6 ± 0.5 | **0.02** |

|  |  |
| --- | --- |

**Supplementary Table S3A.** PbO₂/PaO₂ ratio across PEEP levels in prone position.

Values are reported as median (interquartile range).

|  | | |  | |  | |
| --- | --- | --- | --- | --- | --- | --- |
| PEEP (cmH₂O) | n | | Median (IQR) | |  |  |
| 5 | 8 | | 0.23 (0.15–0.32) | |  |  |
| 10 | 8 | | 0.22 (0.17–0.29) | |  |  |
| 15 | 8 | | 0.20 (0.14–0.31) | |  |  |
| 20 | 8 | | 0.21 (0.13–0.44) | |  |  |

**Supplementary Table S3B.** PbO₂/PaO₂ ratio across PEEP levels in supine position.

Values are reported as median (interquartile range).

| PEEP (cmH₂O) | n | Median (IQR) |
| --- | --- | --- |
| 5 | 8 | 0.28 (0.17–0.48) |
| 10 | 8 | 0.27 (0.15–0.41) |
| 15 | 8 | 0.30 (0.19–0.55) |
| 20 | 8 | 0.29 (0.16–0.60) |

**Supplementary Table S4.** Comparison of intracranial pressure change between Study 1 (healthy lungs) and Study 2 (ARDS lungs).

The change in intracranial pressure (ICP) was calculated as the difference from baseline (PEEP 5 cmH₂O). Values are presented as median (interquartile range). Statistical significance was assessed within and between studies.

| **Parameter** | **Study 1** | **Study 2** |
| --- | --- | --- |
|  | **Healthy lungs** | **ARDS-lungs** |
| ICP-change at PEEP 10 | 0.93 mmHg (0.48 – 1.27) | 0.75 mmHg (-0.21 – 1.51) |
| ICP-change at PEEP 15 | 2.08 mmHg (1.29 – 2.86) | 1.92 mmHg (1.18 – 3.57) |
| ICP-change at PEEP 20 | 3.20 mmHg (1.95 – 4.17) | 3.88 mmHg (2.47 – 7.33) |
| Statistical significance (within study) | Yes (p < 0.01) | Yes (p < 0.01) |
